# Inclusivity in health professional education: how can virtual simulation foster attitudes of inclusion?

**DOI:** 10.1186/s41077-024-00290-7

**Published:** 2024-05-01

**Authors:** Amanda K. Edgar, Joanna Tai, Margaret Bearman

**Affiliations:** 1 Deakin Learning Futures, Deakin University, Melbourne, Australia; 2 School of Education, Faculty of Arts and Education, Deakin University, Geelong, Australia; 3 Centre for Research in Assessment and Digital Learning, Deakin University, Melbourne, Australia

**Keywords:** Inclusive education, Virtual simulation, Health professional education, Educational technology

## Abstract

Disparities in accessing quality healthcare persist among diverse populations. Health professional education should therefore promote more diversity in the health workforce, by fostering attitudes of inclusion. This paper outlines the potential of virtual simulation (VS), as one method in a system of health professional education, to promote inclusion and diversity. We conceptualise how VS can allow learners to experience an alternative to what HPE currently is by drawing on two social justice theorists, Paulo Freire, and Nancy Fraser and their ideas about ‘voice’ and ‘representation’. We present two principles for VS design and implementation: (1) giving voice to learners has the power to transform; and (2) representation in VS builds inclusion. We provide practical means of building voice and representation into VS learning activities, followed by an example. Purposeful and thoughtful integration of these principles paves the way for a more diverse and inclusive healthcare workforce.

## Introduction

Increasing diversity and inclusion in health professional education (HPE) enhances society [1]. In particular, a more diverse workforce with attitudes of inclusion can help address persistent inequality of health outcomes across diverse populations [1–4]. While participation in HPE programs from diverse cohorts is widening, there remains a marked and concerning differential in the levels of attainment [3, 5]. The onus is therefore on HPE programs to promote attitudes of inclusion through the pathway from enrolment to employment [2, 3, 6]. This paper proposes that virtual simulation (VS), when designed with a social justice lens, can offer immediate opportunities for learners to represent their own diverse worlds, and hence foster inclusive attitudes.

Learners who are different may face prejudice from their educational communities and be inhibited from accessing the curriculum [3, 7]. Moreover, learners perceive the disclosure of any personal differences that deviate their image from the ideal can potentially impact their employability [8]. In response to stereotypes, learners may be conscious of appearing ‘normal’ on clinical placements, which may exert undue influence on shaping their professional identity and clinical practice [9].

HPE programs struggle to ensure that clinical placements are environments that promote diversity and inclusion [9, 10]. Often, social practices in these environments can be unsupportive and disabling for learners, coupled with the environment itself being inflexible to the needs of learners or inaccessible [11, 12]. Simulation offers an alternative: experiential learning without the messiness of the clinical environment [13]; this messiness may also limit diversity and impede inclusion [14]. We suggest that VS is particularly valuable due to its scalability and its ability to reach across contexts. In particular, when designing from a social justice perspective, VS offers an opportunity to both be more inclusive and to promote inclusive attitudes.

We define VS to be computer-mediated clinical scenarios where the learner interacts to impersonate the role of a healthcare practitioner within patient narratives. The learner engages through text, selection, movement, or speech while the computer technology creates a sense of presence and immersion [15]. The intention is to allow learners to participate in active learning through deliberate practice and reflection [15]. Like other forms of simulation, VS can also allow learners to assume different perspectives [16]. However, technology itself does not always deliver on its promises—education must be designed to make it more inclusive [17, 18]. Furthermore, we caution that VS cannot be used as an overarching solution or even a piecemeal approach to produce inclusive education. Rather VS needs to be integrated alongside appropriate pedagogies and the values of the curriculum.

There are emerging empirical and conceptual examples of how VS in HPE could influence attitudes toward inclusion in the future healthcare workforce [16, 19, 20]. One empirical study proposes a reflective tool to support simulation delivery teams to evaluate personal assumptions and bias within a VS experience [21]. More targeted conceptual work proposes practical processes for considering equality, diversity, and inclusion as part of the International Nursing Association of Clinical and Simulation Learning standards for design criteria [22]. There are also empirical studies supporting the content that when learners practice applying skills, knowledge and attitudes in a VS environment that exposes them to diversity, it can have an impact on their assimilation of cultural competency [23–25]. In the health professional context, empirical studies confirm that learners’ understanding of the importance of culture improves when they have the opportunity to interact with a specific culture as part of a VS [25–28].

We build on this previous work to explore how social justice theories, particularly the work of Paulo Freire and Nancy Fraser, can frame the design of VS. Critical pedagogies shift the focus of HPE from routine skill development to include reflection and interpersonal engagement as it positions HPE within a wider frame of equality and social justice [29, 30]. Freire’s work has underpinned discussions on the radical repositioning of medical education [30, 31]. Fraser’s ideas form a consistent thread for scholars to explore how social justice might be conceptualised in HPE [32, 33]. We use these theories to explore how VS can provide the means for learners to experience an alternative to HPE as it currently is, through the concepts of voice and representation. By using these radical frames, we introduce profound concepts where we aim with hope and enthusiasm towards a more socially just healthcare workforce.

### Principles for transformation and representation

We describe two theories of social justice that guide our approach. Firstly, we turn to Freire’s seminal work *‘Pedagogy of the Oppressed’* which addresses how education practices uphold the oppressive organisation of society. Freire suggests that education has the capability to radically *transform* society if it can step aside from perpetuating existing social structures [34]. Through education, the structurally and socially disadvantaged (“the oppressed”) can be enabled to reclaim their humanity, and in the process not just liberate themselves but also humanise those that are oppressors [34]. According to Freire, this process of liberation cannot come from a top-down approach and should involve lived experiences where people come together to share dialogue, represent their own realities, and work towards liberation [34]. We suggest that these moments of coming together, where individuals can *voice* their diverse circumstances, can be considered moments of *transformation*.

Our second position takes the observations from Nancy Fraser, a feminist and poststructural social theorist. Fraser suggests patterns that prevent participation are a result of systematic injustice within society. She advocates that transformative strategies correct this injustice by restructuring underlying structures that appear to be the root causes [35, 36]. Her proposals for a more socially just society encompass three dimensions: redistribution, recognition, and representation [35]. *Redistribution* focuses on changing economy-based structures in society that create class, while *recognition* concentrates on shifting status that is created by cultural values in society [35, 36]. The third dimension, *representation*, focuses on changing political patterns that limit the impact that diverse *voices* have on the decision-making process [35]. This is the dimension most relevant to education. When particular people are excluded from fair representation Fraser terms this injustice as *misrepresentation* [35, 37]. More so, those who suffer the injustice of *misframing* are subject to a deeper level of exclusion—they are deemed not to count [35]. According to Fraser, the absence of equal voice, gives rise to misrepresentation and misframing, while the allocation of equal voice—and thus fair representation—opposes situations of wrongful exclusion [35].

Our view is that HPE courses must broaden the notion of inclusion beyond merely granting access to the programs, to allow diverse learners means of fully participating in curriculum. VS provides a valuable means of supporting new pedagogies for full participation. VS alone cannot ‘transform’ healthcare practice, but we suggest that we must confront the problem with action [34]. The transformation to more inclusive forms of HPE, not just *what* should be taught but also *how* things should be taught, and how VS can support this is discussed alongside these social justice frames. We call for the following principles to underpin VS based on the ideas of voice and representation.

### Principle 1: giving voice to learners has the power to transform

VS should offer opportunities for learners to voice their own experiences, perspectives, and concerns. Freirean scholarship suggests that learners must be active participants in their own learning journey and Fraser emphasises equal voices across diverse populations to prevent exclusion [34]. With VS, the diverse features of the technology can enable learners to engage through various forms of participation, for example allowing the choice of learners to make audio or text contributions, thereby allowing for their voice to be included [38]. Learners who might not have had voice and learners who did not previously have access to diverse perspectives therefore have an opportunity to be changed through voice and reflection on voice.

In HPE, it is often assumed that the workplace ‘trumps’ all. However, if learners uncritically adopt the realities of the conditions within the healthcare workforce, then they may lose the ability to imagine the world around them as something that can be transformed. Opportunities for diverse voices within VS could unveil alternatives and allow them to reflect on the limitations of their present social worlds, seeing them as transformable [34]. By allowing the exchange of ideas and experiences, learners’ understanding of the world could be shaped beyond the available clinical placement contexts.

This principle is not just a matter for the learner; it also encompasses those in the facilitation and design of the VS, who in their roles may also be transformed. If the educators act as instigators of transformation themselves they may risk bringing with them their own inherent biases [34]. Instead, by enabling the learner a chance to experience and be heard, designers and facilitators of VS should position themselves as co-learners, learning alongside learners with a pedagogy formed with the learners [34]. In this process, both learners and educators have an opportunity to reflect and gain a consciousness of power dynamics, inequality, and the way in which systems perpetuate injustice [34]. As Freire argues, only once the individual is aware of their own social world will they be able to change it [31].

### Principle 2: representation in VS builds inclusion

VS should foster a sense of belonging if learners encounter their background, culture and self, within the VS environment and are also given opportunities to realistically represent themselves. In HPE, recent studies show learners falsely represent themselves and hide their differences [9, 39]. Fraser suggests that when individuals are required to assimilate the attributes of the majority there is no longer shared respect and equality [36]. Thus VS can inform and restructure social belonging, as simulations themselves can signal who is significant or insignificant [35]. If VS supports realistic representations of learners, then such diversity may foster higher levels of tolerance, friendship, acceptance and understanding [40].

We suggest VS should provide participants with access to different ways of being through exposure to different materials [38]. Whether it is through exchanging ideas and perspectives with peers during a debriefing session or engaging with challenges directly in a simulation activity [38]. This can disrupt social assumptions by leading the learner to embody the authentic narrative/problem in VS, in ways that they might not be able to otherwise access within the curriculum [41]. Thus, the VS can afford learners with new perspectives, which can stimulate reflection and then inspire action upon reality [34, 42]. If thoughtfully designed, VS learning activities therefore may challenge learners’ perceptions of their social worlds, potentially question stereotypes and biases and contribute to acceptance of diversity.

### Towards VS learning activities that promote voice and representation

Exercising thoughtful curation of representation in VS may minimise elements of exclusion but this cannot be done lightly. The oppressors cannot tell the oppressed how things can be transformed and Freire advocates that it is the voice of the individual that should be heard [34]. This challenges traditional education systems and social narratives that may marginalise voice and indeed VS designs that make assumptions about what is represented and how. Biased healthcare practitioners can be the result of misrepresentations and misframings in HPE, impacting patient outcomes and the health of society [37].

Inviting those who belong to different equity groups to review VS content with the aim of understanding if it reinforces negative views and stereotypes may address this. However, to be inclusive, educators must ensure that they are not burdening these populations as they should not be solely responsible for educating others and changing perspectives [43, 44]. Instead we may increase representation through the inclusion of diverse equity groups in learning activities to create environments where marginalised voices are engaged [44]. This may counter misframing and provide an opportunity to make visible to learners the workings of power and inequity. However, if we solely rely on this approach issues of misrepresentation may arise.

Our process needs to recognise that an individual membership to an identity group does not unconditionally represent the knowledge of that group and vice versa [44]. Freire argues that if we stop regarding people in abstract categories and see them as an individual, oppressed and oppressors can genuinely work together [31]. Fraser too recognises that individuals suffer injustice when they are cast into groups [35]. Thereby VS learning activities that work towards promoting the voice and representation of the individual learner foster a deeper understanding of varied experiences.

Again, VS is not just about the technology and learners; it is also about the educators who design and facilitate learning activities. Therefore, programs must invest in preparing educators to facilitate accurate conversations with learners about the representation of race, racism, ableism, and diversity. Otherwise, the VS may become purportedly ‘culturally inclusive’ resources that instead reproduce stereotypical representations that wash away the complexity and multiplicity of identity. This is important to bear in mind in the context of a healthcare system where it is populated with patients who may be vulnerable to well-intentioned healthcare clinicians, who provide care with unintentional bias or, alternatively, intentional bias [45]. It means that educators working with VS, as well as students could usefully grapple with the challenges of social injustices in our healthcare systems, including social determinants of health, biases, marginalisation, unequal relationships, institutional racism and unexamined privilege [46].

### Building voice and representation into VS pedagogy

Voice allows learners to express themselves and their experiences and take part in dialogue. Equally, representation allows learners to have a presence that can shape perceptions and influence attitudes. In this section, we outline ways in which these principles, centred around the interconnected concepts of voice and representation, can be considered, and add value to VS in HPE. We use a broad framework [47] developed for any simulation setting, profession, and modality in Table 1. Again, we highlight that it is not sufficient to merely use VS; inclusive education needs to be consistently considered and embedded into university practices.

**Table 1:** The opportunities for voice and representation incorporated into a simulation framework

Phase	Principle 1: voice	Principle 2: representation
Preparation	• Educators are trained to have open discussions that give the opportunity voice to learners	• Authentically collaborate amongst industry, learners and society for co-creation • Educators and facilitators have an awareness of misrepresentation and misframing
Briefing	• Educators act as facilitators and co-learners rather than taking a top-down approach • All learners and educators in the learning activity agree that all voices are valued and have thought	• Educators clarify roles so that all involved in the learning activity are afforded the chance to be represented
Simulation activity	• Identify various forms of participation for how to engage and interact • Enable self-expression and self-representation for learners and educators involved in the learning activity	• Allow learners to embody diverse perspectives in the role they have in the learning activity • Use voice to provide exposure to diversity that learners otherwise may not have had
Debriefing and/or feedback	• Educator’s use learner-centric behaviours that value learners as legitimate and influential contributors • Educators generate learners’ appetites to explore concepts of inclusion and diversity	• Clarify who is represented • Acknowledging the existence of all learners and their contributions to the experience • Focus on understanding the presence of diverse perspectives • Train educators to facilitate conversations about unfair distributions, racism, diversity and inclusive attitudes
Evaluation	• Allow learners to articulate their perspectives of needs	• Address any present issues of marginalisation and exclusion
Reflection	• All involved in the learning activity reflect on realities at a distance to allow deconstruction	• Learners and educators involved in the learning activity reflect their perceptions of their social worlds and question stereotypes and/or biases

A well-known Australian framework structures simulation in six phases (Table 1) and we apply this to VS. During the preparation phase, detailed planning of the VS is performed, encompassing activities such as constructive alignment, consultation, setting learning objectives and so on [47]. The briefing (or pre-briefing) phase serves to acclimate both learners and educators to the VS activity such as roles, expectations and equipment that will be used [47]. Debriefing and/or feedback may be offered to participants by peers, educators or VS technology [47]. The evaluation phase can contain summative and/or formative assessment of the VS activity, participants’ performance and learning objectives [47]. Reflection is recognised as occurring throughout the other phases but is also given its own focus for further learning after the VS has concluded [47].

### In practice

Many approaches can be adopted when it comes to applying the guidance we have provided to VS. To illustrate, in Table 2 we consider a hypothetical scenario that involves learners in their third year of an HPE course completing a VS. The learners complete the VS synchronously in small groups as part of a problem-based curriculum with alternating non-VS clinical skills activities. One of the VS involves providing care to a child with attention-deficit/hyperactivity disorder (ADHD). In the VS the child is accompanied by a parent for their appointment with a health professional. This marks the child’s initial appointment. The learning outcomes for this scenario are to address the presenting complaint and formulate management decisions.

**Table 2:** Voice and representation are incorporated into the phases of VS with a scenario based on a child with ADHD attending an appointment in a health professional practice

***Case scenario:***
Your next patient is Johnny, a 6-year-old who presents with his father Elijah. Elijah mentioned this is Johnny’s first appointment and he has ADHD The learning objectives are: • to accurately diagnose and address the presenting complaint • to develop evidence-based management decisions
In the preparation phase, a panel approach ensured realistic representation was included in the design of the virtual simulation. This panel included representatives who were learners at the university (not in this course) that identified as having ADHD through the disability resource centre. The educators were also trained to have conversations about ADHD
During the briefing phase, the educator clarified the roles of all participants in the virtual simulation. The learners were required to acknowledge with the educator that all voices within the learning activity were valued equally. The educator also shared a lived experience as a health professional working with a patient who had ADHD and invited learners to volunteer any lived experiences
The virtual simulation allowed the learner to embody Johnny, Elijah, or a practitioner where they made decisions that influenced the narrative. As the narrative changed it provided feedback to the learners about their decisions. For example, when they embodied the practitioner and spent too much time focusing on interacting with the Johnny, they would miss important information that could have been obtained from asking Elijah questions
The virtual simulation allowed the learner to embody Johnny, Elijah, or a practitioner where they made decisions that influenced the narrative. As the narrative changed it provided feedback to the learners about their decisions. For example, when they embodied the practitioner and spent too much time focusing on interacting with the Johnny, they would miss important information that could have been obtained from asking Elijah questions
The learners performed a self-evaluation using a series of questions and ranked their performance on a Likert scale. They also evaluated the VS learning activity and provided qualitative comments. This allowed learners to voice suggestions for improvement and issues of representation that may have occurred during the VS
Learners were provided with a self-reflection pro forma for gradual mindful realisation. In this process, educators also self-reflected on their experiences and revalued their identity and social worlds

This framework should not be used independently of other simulation pedagogy. Instead, it presents an opportunity to implement the authors’ recommendations for fostering diversity and inclusive attitudes by making meaningful adjustments that support diversity and attitudes of inclusion within VS. Further work is required to incorporate the learners’ perspectives into comprehending how these suggestions can be supported, and further evaluation is needed from the perspective of those who take on the role of facilitators and learners. For example, eliciting an understanding of how facilitators support these learning activities could offer potential benefits, such as enhancing debriefing methodology. Further work should also include how these principles may translate or need adaptation when applied to other forms of simulation and the potential challenges and opportunities that different modalities present for applying these principles.

## Conclusion

These principles suggest a way forward for future research and application of VS in the broader system of HPE to support transformation with the goal of inclusion. We have suggested VS as one avenue to incorporate the promotion of voice and representation and address the complex challenges for HPE to incorporate inclusive pedagogies. If learners who graduate from these programs carry forward developed inclusive attitudes and diverse learners progress to professional practice, there is a chance that they can better serve the unmet needs of diverse communities. This itself could lead to a more socially just healthcare workforce and, though VS is not a solution for all challenges, contribute to broader systematic changes in HPE required to move towards these goals. There is work now needed to understand how these principles impact the learning journey and their enduring influence.

## Data Availability

Not applicable.

## Abbreviations

HPE: Health Professional Education
VS: Virtual simulation
ADHD: Attention-deficit/hyperactivity disorder
